# miR-214 Modulates the Growth and Migration of Oral Cancer before and after Chemotherapy through Mediating ULK1

**DOI:** 10.1155/2022/4589182

**Published:** 2022-06-02

**Authors:** Yongtao Yang, Xiaolan Sun, Minghua Li, Limei Li, Shanshan Wang, Yaomin Zhu

**Affiliations:** ^1^Department of Oral & Maxillofacial Surgery, Shenzhen Stomatology Hospital Affiliated to Shenzhen University, Shenzhen City 518000, GuangDong Province, China; ^2^Department of Stomatology, Shenzhen Traditional Chinese Medicine Hospital, Shenzhen City 518033, GuangDong Province, China; ^3^Central Laboratory, Peking University Shenzhen Hospital, Shenzhen City 518022, GuangDong Province, China; ^4^Department of Oral & Maxillofacial Surgery, Shenzhen Stomatology Hospital Affiliated to Shenzhen University, Shenzhen University, Shenzhen City 518000, GuangDong Province, China

## Abstract

The role of miRNAs as crucial components in carcinogenesis has been well documented. However, whether and how miR-214 influences oral cancer cells' drug resistance remains to be elucidated, and its downstream targets are still under investigation. Hence, this research is aimed at determining miR-214 and ULK1 expression in oral cancer before and after chemotherapy and their correlations with cancer cell growth. Human oral normal epithelial cells and human tongue squamous cell carcinoma CAL-27 cells were cultured to detect miR-214 and ULK1 levels. It was found that before chemotherapy, miR-214 was higher, while ULK1 was underexpressed in CAL-27 cells, versus normal epithelial cells. After chemotherapy, miR-214 decreased obviously in CAL-27 cells, while ULK1 level increased significantly. In addition, autophagy-related genes (Beclin 1, mTOR, and P53) in CAL-27 cells were found to be significantly inhibited before chemotherapy and were obviously increased after chemotherapy. Moreover, to further determine the impacts of miR-214 and ULK1 on oral cancer cell growth after chemotherapy, the two were overexpressed or silenced in CAL-27 cells after transfection. We found that ULK1 could effectively decrease the activity and invasion of CAL-27 cells and increase their apoptosis level, while miR-214 could antagonize its antitumor effect. Therefore, miR-214 can be used as an early prognostic biomarker for oral cancer, and ULK1 is a new candidate therapeutic target.

## 1. Introduction

As an important way of food intake, the oral cavity is vulnerable to a variety of external stimuli, such as cold and heat, irritants, and pathogenic microorganisms [1]. Long-term adverse stimuli will destroy the steady state of oral mucosa, resulting oral cancer. Relevant studies find that the maintenance of oral mucosal homeostasis is closely related to autophagy [2]. Autophagy is considered to be a self-protection state of the body. It can recover macromolecular substances in cells and convert them into energy by phagocytizing damaged cells, so as to maintain the stability of metabolism and energy in vivo [3]. However, if adverse stimuli continue to stack, exceeding the stable state maintained by autophagy, it will lead to the accumulation of dysplastic cells and the occurrence of cancer [4]. At present, relevant studies consider that autophagy plays a “double-edged sword” role in the progress of oral cancer. At the early stage, the enhancement of autophagy activity is conducive to eliminate the abnormally proliferating cells in vivo, so as to inhibit tumorigenesis [5]. Then, during cancer progression, autophagy activity can assist the growth and development of cancer cells under the environment lacking blood and oxygen supply [6]. Therefore, autophagy plays an opposite role in different stages of tumor development. In addition, as one of the important auxiliary means in oral cancer therapy, chemotherapy plays an indispensable role. However, in the actual treatment process, chemotherapy drug tolerance will occur, which will reduce the clinical treatment effect. Some studies have shown that autophagy activity will affect the tolerance of tumor cells to chemotherapeutic drugs, thus affecting patients' prognosis [7]. Therefore, the study on the regulation of autophagy in patients with oral cancer is of great significance to explore new therapeutic targets and improve patients' prognosis.

As a noncoding RNA in vivo, the function of microRNA (miRNA) in vivo has gained growing attention from researchers. It is found that miRNA can combine with target genes to regulate the production of functional proteins in vivo, so as to affect the proliferation of many cells. If the regulation is abnormal, it will have an adverse impact on body function. At present, a variety of miRNAs closely related to the progression of oral cancer have been found. For example, miR-203 upregulation will inhibit Tca8113 activity and invasiveness [8], while miR-214 is overexpressed in oral cancer cells and of great significance for their growth and migration [9]. According to the predicted bioinformatics epitope, ULK1 may be the target of miR-214, and it is closely related with autophagy. Autophagy can be divided into four stages: initiation-extension nucleation-maturation and degradation-regeneration [10]. As a homologue of Atg1, ULK1 is closely related to the formation of autophagosomes before the initiation stage [11]. In mouse experiments, if ULK1 gene is knocked out, its autophagy activity will be strongly inhibited. In vivo, ULK1 mainly regulates the formation of preautophagosome through ULK1 complex and can form the core structure of preautophagosome together with Atg13, Atg31, and Atg29 [12]. Moreover, in Ma's study, miR-214 deletion from renal proximal tubules was found to prevent ULK1 expression decline and autophagy defects in diabetic kidneys, thereby reducing nephromegaly and renal proteinuria. However, there has been no study on miR-214/ULK1 axis in oral cancer. Therefore, we speculate that miR-214 may initiate ULK1 mediated autophagy through downregulation of expression after chemotherapy, resulting in further death of tumor cells. The novelty and the motivation of the study is to clarify this mechanism is of great significance for finding new therapeutic targets for oral cancer.

## 2. Materials and Methods

### 2.1. CAL-27 and Human Oral Epithelial Cell Culture

In this study, CAL-27 (CL-0265, Procell, Wuhan, China) and human oral epithelial cells (CP-H203, Procell) were selected for in vitro experiments. In a 37°C cell incubator (51032124, Thermo Fisher Scientific, USA) with 5% CO_2_, CAL-27 was cultured in CAL-27 cell-specific medium (CM-0265, Procell) and human oral epithelial cells in human oral epithelial cell complete medium (CM-h203, Procell), both for 24 h. The cultured CAL-27 cells were assigned to control group and chemotherapy group, among which, cisplatin (10 *μ*g/mL, 100 *μ*L) [13] and paclitaxel (8 *μ*g/mL, 100 *μ*L) [14] were added to the culture medium of chemotherapy group. Eventually, the two groups of cells were cultured in cell incubator for another 24 h.

### 2.2. CAL-27 Cell Transfection

CAL-27 cells in control group cultured in 2.1 were selected as the study cell line and transfected. miR-214 mimic (miR10004564-1-5, RiboBio, Guangzhou, China) and ULK1 mimic (siG082199294701-1-5, RiboBio) were used for overexpression of miR-214 and ULK1, and ULK1 inhibitor (stB0005025a-1-5, Ribobio) was used for silent expression of ULK1. This was followed by transfection with Lipofectamine 2000 purchased from Invitrogen, USA. After six hours of transfection, CAL-27 cells were assigned to control, H-ULK1, L-ULK1, and H-miR-214/H-ULK1 groups. Then, the above cells in each group were diluted to the concentration of 1 × 10^6^/mL; 1 mL cell dilution was taken from each group and dropped into the medium containing cisplatin and paclitaxel. Eventually, cells in all the four groups were treated with 24 h of culture in the cell incubator.

### 2.3. Detection of miR-214 and ULK1 in Cancer Cells by PCR

Beclin 1, mTOR, and p53 are upstream and downstream regulatory signals of autophagy activity, and their levels can indirectly reflect the degree of autophagy activity [15–17]. CP-H203 and CAL-27 cells in control and chemotherapy groups cultured in 2.1 were selected for detection, and the levels of miR-214, ULK1, Beclin 1, mTOR, and p53 in the above cells were detected by PCR. TRIzol reagent isolated total RNA from cells following the instructions (Life Technologies, Thermo Fisher Scientific). qRT-PCRs of mRNAs were reverse-transcribed using the Prime Script RT Master Mix (Applied Biosystems, Foster City, CA), and reactions on the obtained cDNAs were conducted on the ABI StepOneTM Real-Time PCR System (Applied Biosystems, Foster City, CA). See Table 1 for primer information. Transcription alterations relative to *β*-actin or U6 were assessed using 2^−ΔΔCt^.

### 2.4. Activity Evaluation of CAL-27 Cells

CAL-27 cell activity can reflect the efficacy of chemotherapeutic drugs and was detected by immunofluorescence. Ki-67 antibody (ab228549, Abcam, USA) was used to label the active cells in each group cultured in 2.2. After incubating in the dark at 4°C overnight, Alexa fluor 488 (Cy2, FITC) fluorescent secondary antibody was added and incubated without light under 26°C for 40 min. Cell activity was detected by confocal microscope (FV3000, Olympus, Japan).

### 2.5. CAL-27 Cell Apoptosis Evaluation

CAL-27 cell apoptosis was analyzed after transfection to assess the effect of autophagy on chemotherapy. Cells in each group cultured in 2.2 were fixed with stationary solution. After 3 times of PBS cleaning, 0.2% Triton™ X-100 (X100, Sigma-Aldrich, USA) was added to cells and then incubated under 37°C for 10 minutes. Eventually, after cleaning with PBS for another 3 times, the apoptotic cells were labeled with one-step TUNEL Assay Kit (AF647, Wuhan Elabscience Biotechnology Co., Ltd., China). After a 25-minute reaction in a dark environment, cell apoptosis was observed by confocal microscope.

### 2.6. CAL-27 Cell Migration Evaluation

The changes in CAL-27 invasion after chemotherapy was assessed by scratch test. Cells in each group cultured in 2.2 were inoculated into 6-well plates, and the total number of cells was 1 × 10^6^. After scratching the cell layer with a pipette gun, CAL-27 cells at the scratch were washed with PBS. After growing in cell incubator for 24 h, the migration of CAL-27 cells was observed by microscope (DM750M, Leica, Germany).

### 2.7. Statistical Methods

Data analysis was made by SPSS 20.0 (IBM, Armonk, NY). The results were analyzed using a *t*-test and recorded as means ± standard deviations, and differences with *P* < 0.05 were considered significant; each test was run in triplicate.

## 3. Results and Discussion

### 3.1. miR-214 and ULK1 Detection before and after Chemotherapy

**Figure 1:**
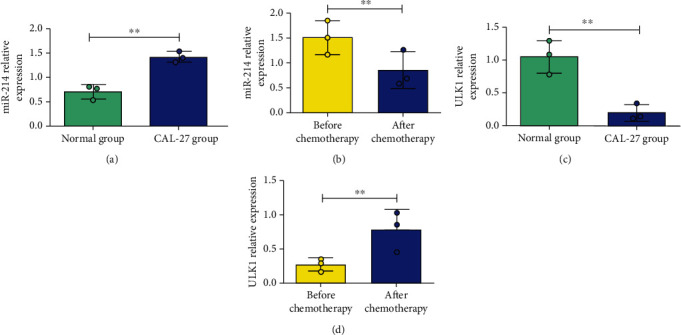
miR-214 and ULK1 detection by PCR. (a) Detection of miR-214 in normal oral epithelial cells and CAL-27. (b) Changes of miR-214 level after chemotherapy. (c) Detection of ULK1 in normal oral epithelial cells and CAL-27. (d) Changes of ULK1 level after chemotherapy. ^∗∗^*P* < 0.01.

miR-214 and ULK1 levels in normal oral epithelial cells and CAL-27 cells were detected, and their changes before and after chemotherapy were observed. It was found that compared with normal oral epithelial cells, miR-214 was overexpressed in CAL-27 cells (Figure 1(a)), and its level decreased significantly after chemotherapy (Figure 1(b)). In addition, ULK1 level was silence expressed in CAL-27 cells (Figure 1(c)), while increased obviously after chemotherapy (Figure 1(d)). miR-214 and ULK1 are associated with the occurrence of oral cancer, and chemotherapy might affect the activity in oral cancer cells by autophagy which needs to be further confirmed by more experiments.

### 3.2. Expression of Autophagy-Related Genes before and after Chemotherapy

**Figure 2:**
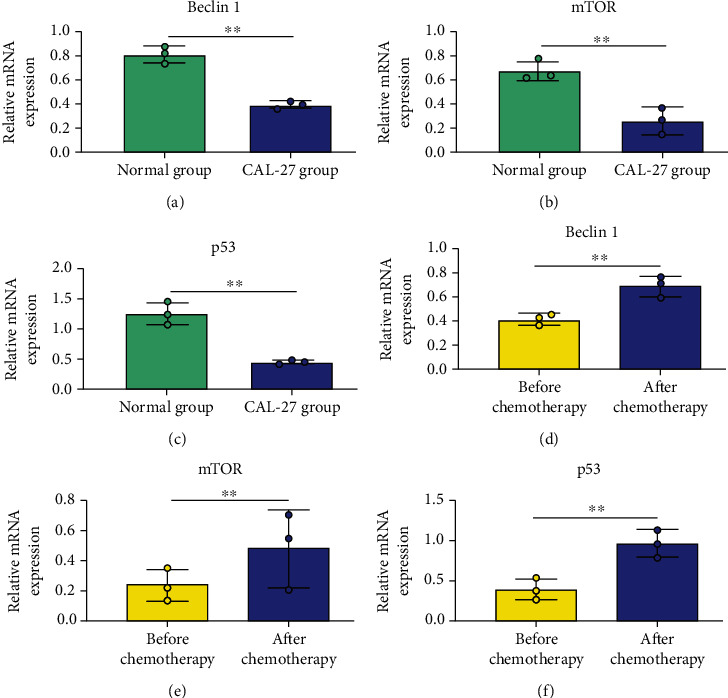
Detection of autophagy activity by PCR. (a) Beclin mRNA expression; (b) mTOR mRNA expression; (c) P53 mRNA expression; (d) changes of Beclin1 mRNA expression after chemotherapy; (e) changes of mTOR mRNA expression after chemotherapy; (f) changes of P53 mRNA expression after chemotherapy; ^∗∗^*P* < 0.01.

Autophagy is an important mechanism leading to the occurrence and development of oral cancer. At the same time, it will also affect tumor cells' sensitivity to chemotherapeutics. Therefore, the changes of autophagy activity in tumor cells after chemotherapy were assessed in terms of autophagy-related genes Beclin1, mTOR, and p53. It was found that the levels of Beclin 1, mTOR, and p53 were obviously reduced in CAL-27 cells compared with normal oral epithelial cells prior to chemotherapy (Figures 2(a)–2(c)), and their levels were significantly increased after chemotherapy (Figures 2(d)–2(f)), suggesting that chemotherapy can promote the expression level of autophagy-related gene in tumor cells.

### 3.3. Growth of CAL-27 Cells after Chemotherapy

**Figure 3:**
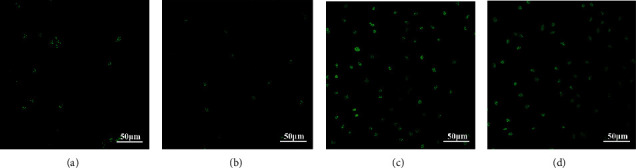
CAL-27 cell proliferation assessment after chemotherapy. (a) Ki67 expression pattern in CAL-27 cells without treatment. (b) Ki67 expression pattern in CAL-27 cells in H-ULK1 group. (c) Ki67 expression pattern in CAL-27 cells in L-ULK1 group. (d) Ki67 expression pattern in CAL-27 cells in L-ULK1/H-miR-214 group.

The growth situation of tumor cells can reflect tumor progression. For the purpose of further exploring the impacts of miR-214 and ULK1 on CAL-27 cell growth, we labeled surviving cells after chemotherapy with Ki-67 antibody. It was found that CAL-27 cells grew worse in H-ULK1 group (Figures 3(a) and 3(b)). On the contrary, when ULK1 was silenced, or miR-214 was overexpressed and ULK1 was silenced, the growth of CAL-27 cells was greatly improved, especially in the H-miR-214/L-ULK1 group (Figures 3(c) and 3(d)). Therefore, ULK1 can decrease CAL-27 cell activity, and its downexpression can improve the tolerance of tumor cells to chemotherapy, while miR-214 can antagonize its effect on tumor cell proliferation.

### 3.4. Apoptosis Evaluation of CAL-27 Cells after Chemotherapy

**Figure 4:**
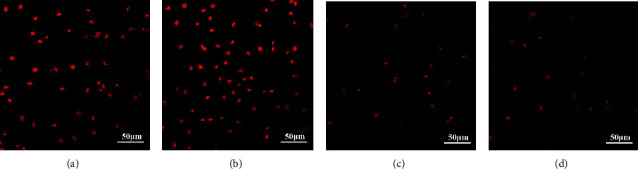
CAL-27 cell apoptosis detection after chemotherapy. (a) Apoptosis level of CAL-27 cells without treatment. (b) Apoptosis level of CAL-27 cells with ULK1 overexpression. (c) Apoptosis level of CAL-27 cells with ULK1 downexpression. (d) Apoptosis level of CAL-27 cells with ULK1 downexpression and miR-214 overexpression.

The apoptosis degree can indirectly reflect the proliferative activity of tumor. After chemotherapy, compared with control group (Figure 4(a)), more apoptosis cells were found in H-ULK1 group (Figure 4(b)), while the apoptosis rate in L-ULK1 group was less (Figure 4(c)), especially in the H-miR-214/L-ULK1 group (Figure 4(d)). The above results suggest that miR-214 can antagonize the effect of ULK1, thus inhibiting the CAL-27 apoptosis.

### 3.5. Migration Evaluation of CAL-27 Cells after Chemotherapy

**Figure 5:**
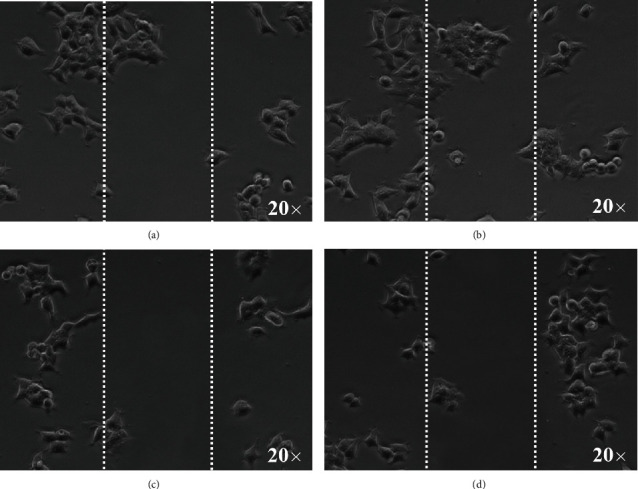
Scratch test of CAL-27 cells after chemotherapy. (a) CAL-27 cells without any treatment, 20x; (b) CAL-27 cells with ULK1 silence-expression, 20x; (c) CAL-27 cells with ULK1 overexpression, 20x. (d) CAL-27 cells with miR-214 overexpression and ULK1 silence-expression, 20x.

Tumor cell migration was detected by scratch test, so as to evaluate the invasiveness of tumor cells after chemotherapy. Compared with control group (Figure 5(a)), Figure 5(b) shows that more CAL-27 cells migrate to the scratch area in the L-ULK1 group, while CAL-27 cells remain more stable in the H-ULK1 and H-miR-214/L-ULK1 groups (Figures 5(c) and 5(d)), which means that miR-214 can better antagonize the effect of ULK1 and reduce the migration of CAL-27 cells.

## 4. Discussion

The role of miRNA in vivo has been found to be more and more prominent with the deepening of research. Its abnormal expression will cause multiple tumors and can be used as a potential marker for tumor prognosis and therapy. For example, miR-214 can inhibit the activity of hepatocellular carcinoma by targeting E2F3 [18]. In nasopharyngeal carcinoma, miR-214 can activate Akt signal pathway by regulating PTEN, so as to promote the proliferation activity of nasopharyngeal carcinoma cells [19]. Relevant studies have shown that miR-214 level increases significantly in a variety of tumors and can regulate Wnt/*β*-catenin signaling pathway to promote cancer cell growth [20–22]. However, studies on the correlation between miR-214 and autophagy regulation are few. In addition, during chemotherapy, the emergence of drug resistance of cancer cells is closely related to autophagy. It can help cancer cells better obtain energy supply, thus helping them tolerate the adverse proliferation environment of ischemia and hypoxia and increasing their tolerance to chemotherapeutic drugs. However, autophagy will also promote the apoptosis of tumor cells, which is not conducive to their proliferation. Therefore, to study the correlation between miR-214 and ULK1 mediated autophagy and to explore their effects on tumor cell proliferation after chemotherapy is of great implications for oral cancer diagnosis and therapy.

miR-214 level in oral epithelial cells was found lower than that in CAL-27 cells; on the contrary, ULK1 level increased significantly. In addition, miR-214 level in CAL-27 cells decreased significantly after chemotherapy, while ULK1 level increased significantly. The results was similar to a previous study [23], which reported markedly downregulated miR-214 in colorectal cancer cell lines and blood of CRC patients after irradiation exposure. Except from these, miR-214 was also found to be underexpressed in 5-FU-resistant colon cancer cells compared to normal cells and could sensitize nonresistant and 5-FU-resistant colon cancer cells to 5-FU in vitro.

To better explore the function of miR-214 in oral cancer progression and its correlation with ULK1, as well as the effect of autophagy on chemotherapy, we carried out experiments in CAL-27 cells. It showed that when ULK1 was low expressed during chemotherapy, the autophagy activity in CAL-27 was greatly inhibited, while the activity of tumor cells increased significantly. It was more prone to invasion and metastasis, so as to accelerate the development of oral cancer and improving the tolerance to chemotherapeutic drugs, thus leading to poor prognosis. In addition, the silent expression of miR-214 negatively regulated ULK1 level, thereby increasing autophagy activity in CAL-27 cells, leading to tumor cell apoptosis and inhibiting distant metastasis. Autophagy is an important part of physiological activities and is considered to be a protective mechanism of the human body. The cells in vivo decompose heterotypic proliferating cells through self-phagocytosis, thus completing the recovery and conversion of energy, which is of great significance for realizing cells' self-renewal and maintaining the stability of the internal environment [24, 25]. However, if the activity of autophagy changes or the regulation of autophagy signal pathway is abnormal, it will have an adverse impact on the physiological function of the body and lead to the occurrence of cancer [26]. For example, autophagy-related gene Beclin 1 decreases in breast cancer, and its downregulation will promote epithelial mesenchymal transition and reduce the inhibitory effect on EGFR [27], thus promoting the growth and migration of breast cancer. Otherwise, as a key gene regulating autophagy, Atg15 plays a dynamic role in regulating autophagy in hepatocellular carcinoma development, and it can inhibit Wnt/*β*-catenin signaling pathway, thereby inhibiting the malignant biological behavior of hepatoma cells [28]. Similarly, ULK1 plays a key role in the formation of pre autophagosome and promoting the occurrence of autophagy and has been found to be abnormally expressed in a variety of malignant tumors.

Therefore, miR-214 can be used as an early biomarker of oral cancer, and its increase will promote the progress of oral cancer. Otherwise, ULK1 can regulate autophagy in oral cancer and can be used as a new potential target to evaluate the prognosis of oral cancer and improve chemotherapeutic effect.

## 5. Conclusion

This paper studied miR-214 and ULK1 levels in oral cancer and discussed their effect on chemotherapy through regulating autophagy. It revealed overexpressed miR-214 in CAL-27, suggesting that miR-214 could promote oral cancer progression. On the contrary, ULK1 level decreased significantly, thereby effectively inhibiting the activity and migration of CAL-27 cells and promoting their apoptosis levels. After chemotherapy, miR-214 level was significantly inhibited, while ULK1 level was obviously increasing. In addition, in the process of chemotherapy, the low level of ULK1 will inhibit autophagy activity in tumor cells, which will increase their tolerance to chemotherapeutic drugs, so as to promote the invasion and growth of cancer cells. However, when miR-214 is overexpressed, it could antagonize the inhibitory effect of ULK1 on tumor. Therefore, for oral cancer, miR-214 can be an early prognostic biomarker while ULK1 is a new candidate therapeutic target. However, there are still limitations in this study, which is very inadequate in proving that miR-214/ULK1 axis is related to autophagy, and there is a lack of evidence to prove that miR-214/ULK1 axis is related to autophagy. In addition, the expression of autophagy-related proteins was not detected. Thus, in order to demonstrate the connection between miR-214/ULK1 axis and autophagy, more comprehensive and well-designed experiments are needed in further studies.

## Figures and Tables

**Table 1:** Primers information of PCR.

	F primer	R primer
miR-214	5′-AGCATAATACAGCAGGCACAGAC-3′	5′-AAAGGTTGTTCTCCACTCTCTCAC-3′
ULK1	5′-ACATCCGAGTCAAGATTGCTG-3′	5′-GCTGGGACATAATGACCTCAGG-3′
U6	5′-CTCGCTTCGGCAGCACA-3′	5′-AACGCTTCACGAATTTGCGT-3′
Beclin 1	5′-AGCTCAGTACCAGCGGGAGT-3′	5′-TGGAAGGTGGCATTGAAGAC-3′
mTOR	5′-AGTGGGAAGATCCTGCACATT-3′	5′-TGGAAACTTCTCTCGGGTCAT-3′
P53	5′-ATCCTTACCATCATCACACTGGAA-3′	5′-CAGGACAGGCACAAATACGAAC-3′
*β*-Actin	5′-GGGAAATCGTGCGTGACATT-3′	5′-GCGGCAGTGGCCATCTC-3′

## Data Availability

The labeled dataset used to support the findings of this study are available from the corresponding author upon request.
